# Excision of the Tonsil

**Published:** 1865-04

**Authors:** John T. Meyers

**Affiliations:** Fairmount, Ills.


### EXCISION OF THE TONSIL.

By JOHN T. MYERS, M. D., Fairmount, Ills.

Miss A., aged 18, who had previously enjoyed good health, went to her bed in apparent good health, on the evening of the 13th of February and slept well until about 2 o'clock A. M., when she arose with a very severe pain in the left ear. The pain being so intense, she arose and went to the family room. On entering her mother's room, she told her mother she believed she would vomit, (spoke plainly), and, making a noise as if choking, her father sprang to her relief and found she was choking. He placed his finger in her mouth and detected a large body in the throat that seemed to obstruct the passage of air; he at once pulled it aside, and immediately sent for Dr. Ray, Dr. New and myself. On arriving, we found the right tonsil (held aside by the father) considerably enlarged and elongated, acting without due meditation; and, thinking temporary relief might be obtained, at least to suffice until daylight, I punctured the tonsil with a bistoury, but it did no good; no discharge followed the operation except a few drops of blood. We then decided that excision must be performed or death would ensue. The operation was immediately performed by Dr. Ray, (the tonsil being held by a hook,) with a probe-pointed bistoury.

The young lady was immediately relieved, or sufficiently so to sleep a little before 7 o'clock A. M. The excised portion was at least one-third larger than the egg of a pigeon. No hemorrhage followed the operation and the patient rapidly recovered, with the exception of an abscess of the left ear, which is now well and no defect of the hearing.

March 7th, 1865.

